# HS-GC-IMS Analysis of Volatile Organic Compounds in Different Varieties and Harvesting Times of *Rhizoma gastrodiae* (Tian Ma) in Yunnan Province

**DOI:** 10.3390/molecules28186705

**Published:** 2023-09-20

**Authors:** Hao Duan, Shiqi Zhou, Jinhong Guo, Wenjie Yan

**Affiliations:** 1 College of Biochemical Engineering, Beijing Union University, Beijing 100023, China; 20221083210418@buu.edu.cn (H.D.);; 2 Beijing Key Laboratory of Bioactive Substances and Functional Food, Beijing Union University, Beijing 100023, China

**Keywords:** *Rhizoma gastrodiae*, HS-GC-IMS, identification, different species, cluster analysis

## Abstract

Headspace–gas chromatography–ion mobility spectrometry (HS-GC-IMS) coupled with principal component analysis (PCA) was used to investigate the differences in volatile organic compounds (VOCs) in four different varieties of Yunnan Huang Tian Ma (containing both winter and spring harvesting times), Yunnan Hong Tian Ma, Yunnan Wu Tian Ma, and Yunnan Lv Tian Ma. The results showed that the flavor substances of different varieties and different harvesting times of *Rhizoma gastrodiae* were mainly composed of aldehydes, alcohols, ketones, heterocycles, esters, acids, alkenes, hydrocarbons, amines, phenols, ethers, and nitrile. Among them, the contents of the aldehydes, alcohols, ketones, and heterocyclic compounds are significantly higher than those of other substances. The results of cluster analysis and fingerprint similarity analysis based on principal component analysis and Euclidean distance showed that there were some differences between different varieties of Yunnan *Rhizoma gastrodiae* and different harvesting times. Among them, Yunnan Lv Tian Ma and Wu Tian Ma contained the richest volatile components. Winter may be the best harvesting season for Tian Ma. At the same time, we speculate that the special odor contained in Tian Ma should be related to the aldehydes it is rich in, especially benzene acetaldehyde, Benzaldehyde, Heptanal, Hexanal, Pentanal, and butanal, which are aldehydes that contain a strong and special odor and are formed by the combination of these aldehydes.

## 1. Introduction

**Figure 1:**
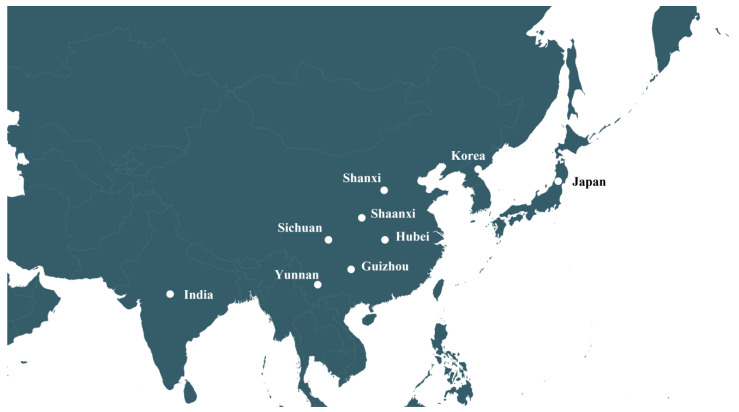
Schematic representation of the main distribution areas of *Rhizoma gastrodiae*.

The main areas of origin and consumption of *Rhizoma gastrodiae* (Tian Ma) are in Asian countries [1], and its main distribution areas are shown in Figure 1. In many areas of China, *Rhizoma gastrodiae* is used by local people as a healthy and tasty food in stews, hot pots, and stir-fries [2]. Currently, it is considered that the quality of Tian Ma from Yunnan, China is better and is well-recognized by consumers and the market. *Rhizoma gastrodiae* can be broadly classified into five species based on the different colors of the flowers, the flower stems, the shape of the tubers, and the water content of the tubers, including Wu Tian Ma (*Gastrodia elata* Bl. f. *glauca* S. Chow), Hong Tian Ma (*Gastrodia elata* Bl. f. *elata* ex S. Chou et S.C. Chen), Huang Tian Ma (*Gastrodia elata* Bl. f. *flavida* S. Chow), Lv Tian Ma (*Gastrodia elata* Bl. f. *viridis* Makino), and Song Tian Ma (*Gastrodia elata* Bl. f. *alba*). Among them, the species currently cultivated on a large scale are mainly Hong Tian Ma, Wu Tian Ma, and various hybrids of Tian Ma, while Song Tian Ma and Lv Tian Ma are very rare and not cultivated on a large scale, and Song Tian Ma is barely circulating in the market. Therefore, all varieties of Tian Ma except Song Tian Ma were collected and tested in this study. Studies have shown that there are significant differences in the chemical composition and pharmacological activity between different varieties of *Rhizoma gastrodiae* [3].

Not only is *Rhizoma gastrodiae* a food product, but it is also a medicinal plant with a wealth of active substances and health functions. Studies have shown that *Rhizoma gastrodiae* is rich in gastrodin, p-hydroxybenzyl alcohol, parishin, polysaccharides, sterols, organic acids, and other components [4], which also makes *Rhizoma gastrodiae* play a better protective role in the central nervous system and can significantly improve diseases such as Alzheimer’s disease, vascular dementia, epilepsy, Parkinson’s disease, and cerebral ischemia/reperfusion [5,6,7]. As a result, *Rhizoma gastrodiae* has greater potential for development in the functional food and pharmaceutical markets, and *Rhizoma gastrodiae*-related functional foods are gradually appearing on the market and receiving attention from researchers and consumers alike. Therefore, research on the flavor of *Rhizoma gastrodiae* is particularly important if we want to develop functional foods that are satisfactory to consumers. However, some studies have shown that different origins and varieties have a greater impact on the active ingredients in fresh *Rhizoma gastrodiae* [8] and further influence the extent to which *Rhizoma gastrodiae* can perform its health functions. Also, differences in the volatile components of *Rhizoma gastrodiae* are among the most important factors affecting consumer acceptance. Flavor determines the organoleptic value of a food product and plays an important role in the identification of its nutritional value. Therefore, in this paper, four varieties of Yunnan *Rhizoma gastrodiae*, which are more widely distributed in the market and have two harvest seasons, were selected for the analysis and study of flavor components.

In recent years, there has been an increasing amount of research into the use of instrumental analytical techniques for the detection of volatile flavor components in food, which can give more objective analytical data on the substance being tested at the molecular level, complementing sensory analysis. Commonly used instrumental analytical techniques are the electronic nose (E-nose), gas chromatography–mass spectrometry (GC-MS), and gas chromatography–ion mobility spectrometry (GC-IMS). Of these, the E-nose is an intelligent system with rapid detection and high sensitivity [9]. GC-IMS has the advantage of high sensitivity for the characterization of volatile compounds, and it and E-nose are also widely used to distinguish authentic and adulterated samples because of their ease of operation [10,11,12]. However, the reproducibility of E-nose assay results is slightly lower and has limitations in some assays [13,14]. Studies have shown that GC-IMS is fast, sensitive, and easy to use [15]. In addition, GC-IMS has outstanding high separation efficiency compared to GC-MS, allowing analytical results to be obtained in a shorter period, and the 3D spectral results include the retention time, drift time, and signal intensity, making qualitative analysis more accurate [12]. In recent years, GC-IMS has been widely used for the study of volatile compounds in food and functional substances [15], e.g., shiitake mushrooms [16], green tea [17], and gum [18]. GC-IMS has also been used more frequently in the field of food analysis in recent years and is developing rapidly. It has the advantages of a small sample size, simple operation, high sensitivity, and accurate results, and it is suitable for the characterization of volatile compounds with various properties [19,20]. Very few studies have been carried out using HS-GC-IMS for the detection and analysis of volatile components in *Rhizoma gastrodiae* samples, and Sun et al. used GC-IMS to compare and analyze the differences in volatiles in fresh *Rhizoma gastrodiae* from different species and origins, which is a convenient and simple method to construct [8]. In the same way, Qiu et al. used it for the detection of volatile chemical components in dried *Rhizoma gastrodiae* in Guizhou and found that the method is simple, rapid, accurate, and has a small sample size [21]. However, studies on several species of dried *Rhizoma gastrodiae* have not been reported. Moreover, Qiu et al.’s study also tested and analyzed only one type of *Rhizoma gastrodiae* in Guizhou and did not further analyze the flavor variability of multiple varieties of *Rhizoma gastrodiae* in the region.

The richest source of *Rhizoma gastrodiae* is mainly in China, and Yunnan *Rhizoma gastrodiae* is commonly recognized in the Chinese *Rhizoma gastrodiae* trade, which may be related to the abundance of *Rhizoma gastrodiae* varieties of Yunnan origin and the maturity of local standardized cultivation and processing techniques. Currently, there are fewer studies on the variability of volatile flavor substances between different varieties of dried *Rhizoma gastrodiae* products. However, different varieties and harvesting times have a greater impact on the volatile components in *Rhizoma gastrodiae* and further influence the flavor and nutrition of the product. Therefore, during this comparative study, we compared Yunnan Huang Tian Ma (containing both winter and spring harvesting times), Yunnan Hong Tian Ma, Yunnan Wu Tian Ma, and Yunnan Lv Tian Ma, which are the five *Rhizoma gastrodiae* species that differ in their volatile organic matter. It is hoped that this will provide some reference value for the processing and nutritional value of *Rhizoma gastrodiae* functional foods.

## 2. Results

### 2.1. Analysis of Volatile Components in Different Rhizoma gastrodiae Samples by HS-GC-IMS

**Figure 2:**
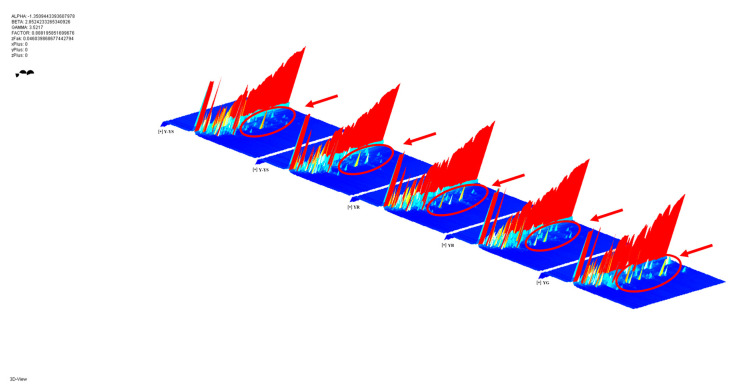
Three 3D topographic plots.

HS-GC-IMS was used to analyze the VOCs in five samples of *Rhizoma gastrodiae*. Figure 2 shows the 3D spectra generated by HS-GC-IMS, which contains data such as the retention time, drift time, and peak intensity, and different peak intensities are represented by the different sample VOCs. It can be visually observed in Figure 2 that there is some variation in the volatile chemical composition of the different *Rhizoma gastrodiae* samples. The differences in the peak intensities between the different species are circled in red in Figure 2.

**Figure 3:**
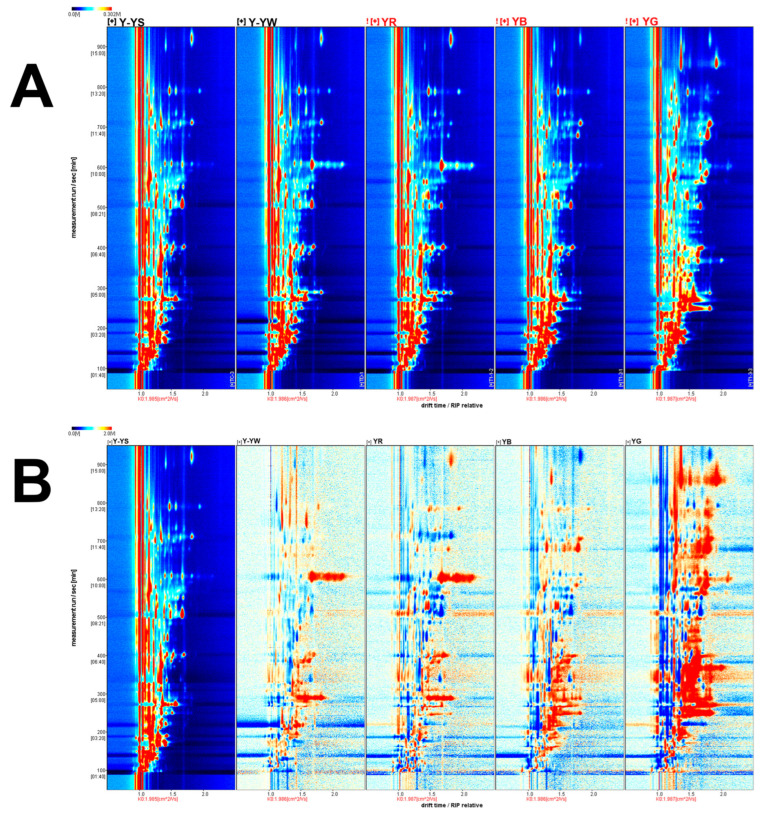
Topographic map of the five *Rhizoma gastrodiae* samples (**A**) and a comparative map of the differences between the five *Rhizoma gastrodiae* samples (**B**).

The 3D spectrograms enabled an initial, but rather crude, observation of the general variability between the samples. Therefore, a 2D top view of the VOCs in the five *Rhizoma gastrodiae* samples was plotted using the Reporter plug-in (Figure 3), enabling a detailed comparison of the differences in VOCs between the different *Rhizoma gastrodiae* samples. The vertical coordinate indicates the retention time of the GC, and the horizontal coordinate indicates the drift time and the reactive ion peak (RIP.) The different-colored points on either side of the RIP represent the different VOCs detected. As can be seen from Figure 3A, most of the signal occurred at retention times of 80–970 s and drift times of 1.0–1.9 ms. And the difference in color on either side of the peak elucidates the signal intensity of the different volatiles detected in each *Rhizoma gastrodiae* sample. The red signal indicates a higher concentration of the detected volatiles, and the darker the color, the higher the intensity, indicating a higher concentration of that volatile substance. The white color indicates lower concentrations of the detected volatiles.

A differential contrast model was used to compare the differences between samples, using the Y-YS sample as the reference contrast and the remaining sample minus the reference. If the two VOCs agree, the background after subtraction is white; if it is red, it means that the substance concentration is higher than the reference, and blue means it is lower than the reference. In the differential contrast model plot (Figure 3B), the concentrations of the volatile substances can be seen. As can be seen in Figure 3B, there were large differences in the volatiles in the samples of *Rhizoma gastrodiae* of different species and different harvesting times. Comparing the samples Y-YS, Y-YW, and YR, in the range of 290–310 s for the retention time and 1.3–1.8 ms for the drift time, Isovaleric acid, Hexanal, 2,3-Butanediol, and the concentrations of Octanal and 3-Octanol were significantly higher in Y-YW and YR than in Y-YS in the range of 590–610 s for the retention time and 1.7–2.3 ms for the drift time. For 1.3–1.8 ms, the VOC concentrations of 2-heptanone, Heptanal, n-Hexanol, pentanoic acid, N-nitrosodiethylamine and (E)-2-hexenal were significantly higher in YB than in Y-YS. A comparison of the samples Y-YS and YG showed that the concentration of volatile components in the sample YG was significantly higher than that in the sample Y-YS. These data differences indicate that there is a greater variation in the volatile components of different varieties of *Rhizoma gastrodiae*. This also indirectly suggests that under the present experimental conditions, Lv Tian Ma contains more flavor substances compared to other regions, which explains why it has a more pronounced aroma in all samples.

Previously, Sun et al. used GC-IMS to detect the differences in volatile components between three fresh *Rhizoma gastrodiae* varieties, namely, Hong Tian Ma, Wu Tian Ma, and the hybrid *Rhizoma gastrodiae*, and found that Wu Tian Ma was richer in volatile components than the two varieties of *Rhizoma gastrodiae* [8]. Meanwhile, Cao et al. conducted a comparative study on the volatile components of Wu Tian Ma in different harvesting seasons based on E-nose and GC-IMS and found that winter was the suitable harvesting period for harvesting *Rhizoma gastrodiae* [22]. Our study found that the YG samples contained the highest content of volatile substances and more species, followed by YB, and that winter-harvested *Rhizoma gastrodiae* had a higher content of volatile substances and species compared to spring-harvested *Rhizoma gastrodiae*. Our study’s results are like the results of the previous study. It also shows from the side that under the present experimental conditions, Yunnan Lv Tian Ma and Wu Tian Ma have better quality compared to other origins and species of *Rhizoma gastrodiae*. The winter harvest of *Rhizoma gastrodiae* is its best harvesting period.

### 2.2. Identification of Volatile Components in Different Rhizoma gastrodiae Samples

**Figure 4:**
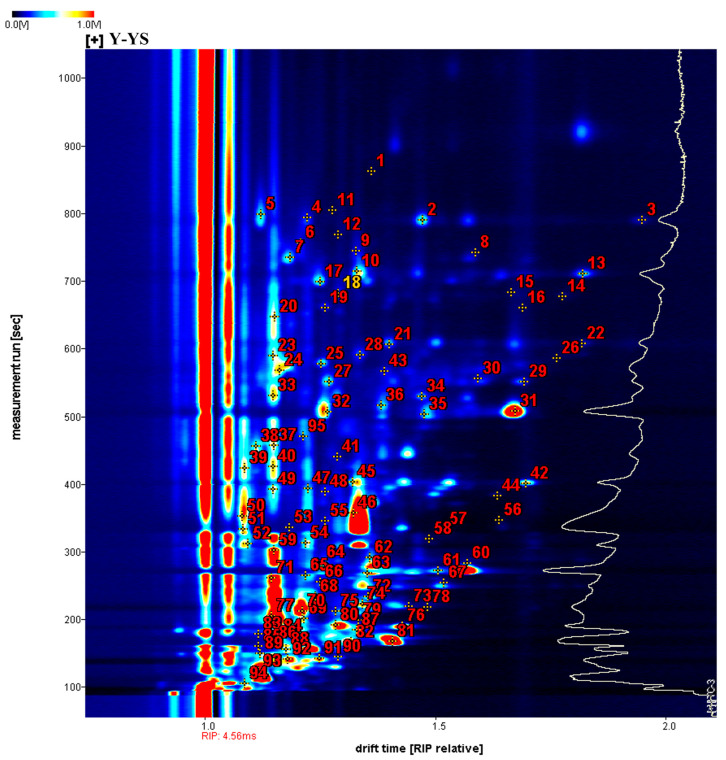
HS-GC-IMS profiles of five samples of *Rhizoma gastrodiae* (the different dots in the figure represent the identified volatile components, and the numbers correspond to the names of the identified volatile components in Table 1).

In this study, HS-GC-IMS was used to analyze the differences in volatile organic compounds in Yunnan Huang Tian Ma (containing both winter and spring harvesting times), Yunnan Hong Tian Ma, Yunnan Wu Tian Ma, and Yunnan Lv Tian Ma, four species, and two harvest times, giving qualitative characterization information. As shown in Figure 4, the horizontal coordinates indicate the differential time, the vertical coordinates indicate the resolution time, and the red numbers correspond to the compounds in Table 1. A total of 160 signal peaks were identified, and 95 compounds were characterized, including 24 aldehydes, 15 alcohols, 14 ketones, 13 heterocyclic compounds, 9 esters, 5 acids, 4 alkenes, 3 hydrocarbons, 3 amines, 2 phenols, 2 ethers, and 1 nitrile. Of these, Nonanal, ortho-Guaiacol, Acetophenone, (E)-2-octenal, Limonene, Octanal, 3-Octanol, 1-octen-3-one, (E)-hept-2-enal, Benzaldehyde ethylpyrazine, Heptanal, 2-heptanone, pentan-1-ol, Hexanal, 3-methylbutanal, and butanal exist in monomeric and dimeric forms.

Previously, Sun et al. used GC-IMS to analyze the differences in volatile compounds in three varieties of fresh *Rhizoma gastrodiae*, and a total of 75 volatile compounds were detected. Of these, 45 substances could be identified, including 16 aldehydes, 9 esters, 6 alcohols, 5 ketones, and 3 acids [8]. Qiu et al. used HS-GC-IMS to identify 25 volatile components in *Rhizoma gastrodiae* and found that the most abundant compounds were aldehydes [21]. Whereas our study similarly found that aldehydes were the most abundant component in *Rhizoma gastrodiae*, we detected a greater abundance of volatile components, presumably because Lv Tian Ma contributed a richer number of volatile components related to this.

### 2.3. Topographic Results and Analysis of Five Rhizoma gastrodiae Samples

**Figure 5:**
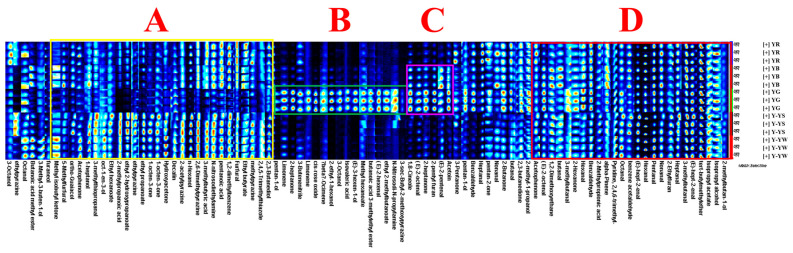
Fingerprints of the five *Rhizoma gastrodiae* samples (**A**–**D**) indicate the four discussion areas selected below the letters).

To compare differences in specific volatiles from each *Rhizoma gastrodiae* sample, the peaks of all samples were selected for fingerprint comparison (Figure 5). Each row of the graph represents the substance detected, and each column represents the signal intensity of the same volatile substance in different *Rhizoma gastrodiae* samples. Each dot represents a volatile substance, the shade of the color represents the level of the detected volatile substance, and the brighter the color, the higher the level.

Methyl isobutyl ketone, 5-methylfurfural, ortho-guaiacol, acetophenone, 1-heptanol, 3-methylthiopropanal, oct-1-en-3-ol, ethyl hexanoate, 2-methylpropanoic acid, ethyl 2-hydroxypropanoate, ethylpyrazine, ethyl propanoate, 1-octen-3-one, hydroxyacetone, decalin, 2-acetylpyrazine, n -hexanol, 2,6-dimethylpyrazine, 3-methylbutyric acid, N-nitrosodiethylamine, pentanoic acid, 1,2-dimethylbenzene, furfural, ethyl butyrate, 2,4,5-trimethylthiazole, and 2,3-butanediol were the characteristic substances of the samples except for Yunnan Lv Tian Ma (Box A).

Pentan-1-ol, limonene, 2-heptanone, 3-butenenitrile, cis-rose oxide, 2-ethyl-l-hexanol, beta-ocimene, 3-octanol, isovaleric acid, (E)-3-hexen-1-ol, methyl hexanoate, butanoic acid 3-methylethyl ester, (E)-2-hexenal, ethyl 2-methylbutyrate, N-Nitrosodi-N-propylamine, and 3-sec-butyl-2-methoxypyrazine are the characteristic substances of Yunnan Lv Tian Ma (Box B).

1,8-cineole, (E)-2-octenal, 2-heptanone, 2-pentyl furan, (E)-2-pentenal, and acetoin are the characteristic substances of Yunnan Wu Tian Ma and Yunnan Lv Tian Ma (Box C).

Once again, the above results show that Yunnan Lv Tian Ma is the richest in volatile organic compounds, followed by aconite. For the species of Huang Tian Ma, winter-harvested *Rhizoma gastrodiae* had more volatile components than spring-harvested *Rhizoma gastrodiae*, and ethylpyrazine was a unique volatile component of spring-harvested *Rhizoma gastrodiae*. Octanal, Butanoic acid methyl ester, and 3-Methyl-3-buten-1-ol are volatile components specific to the winter harvest.

Second, during our research, we found that the dried *Rhizoma gastrodiae* contained a very specific odor. In a previous study, Lv described this odor as “horse urine” and hypothesized that Dimethyl disulfide was involved in the production of this odor [23]. Huang et al. hypothesized that Pyrazine and tetramethyl- were the main causes of these odors in *Rhizoma gastrodiae* [24]. In our study, all five species of *Rhizoma gastrodiae* were found to contain acetophenone, (E)-2-octenal, 1,2-dimethoxyethane, butanal, 3-methylbutanal, 2-hexanone, hexanal, benzaldehyde, 2-methylpropionic acid, alpha-pinene, pyridine,2,4,6-trimethyl-, octanal, benzene acetaldehyde, (E)-hept-2-enal, pentanal, nonanal, 2-ethylfuran, heptanal, tert-butylmethylether, isopropyl acetate, isopropyl alcohol, and 2-methylbutan-1-ol (Box D). Of these 22 volatile components, aldehydes accounted for 50%. Benzene acetaldehyde odors are described as nut and pungent, Benzaldehyde odors are described as almond and burnt sugar, Heptanal odors are described as fat, citrus, and rancid, 2-Hexanone and Hexanal odors are described as grass, tallow, and fat, Pentanal odors are described as almond, malt, and pungent, 2-Ethylfuran odors are described as butter and caramel, butanal odors are described as pungent and green, and alpha-Pinene odors are described as cedarwood, pine, and sharp. Most of these substances are aldehydes, and it is assumed that the odors of *Rhizoma gastrodiae* are related to the aldehydes it contains, especially benzene acetaldehyde, benzaldehyde, Heptanal, Hexanal, Pentanal, and butanal. The combination of several aldehydes with a strong and specific odor was formed.

### 2.4. Cluster Analysis of the Volatile Components of Five Rhizoma gastrodiae Samples

#### 2.4.1. Dynamics of the Sample

**Figure 6:**
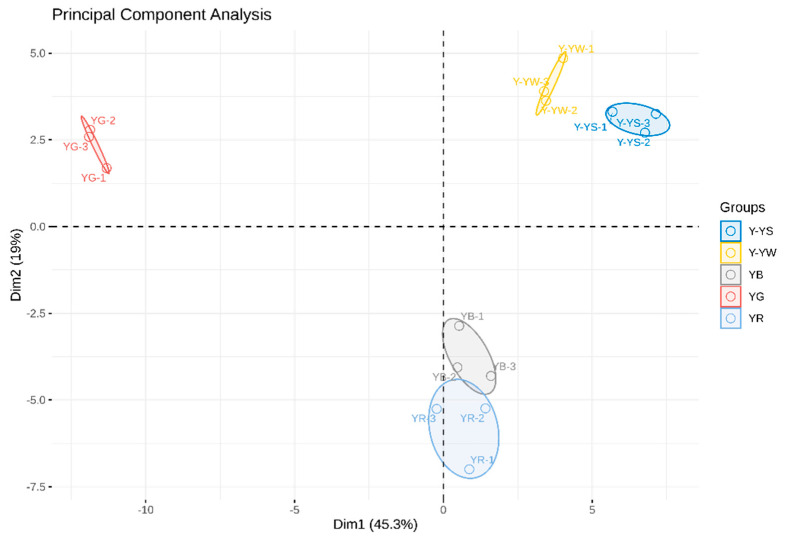
Results of the PCA analysis of five *Rhizoma gastrodiae* samples.

Principal component analysis (PCA) is a multivariate statistical analysis method that transforms the original correlated variables into linearly uncorrelated variables through multiple correlation transformations, providing researchers with a way to reduce the dimensionality of the data, thereby eliminating non-essential information [25,26] and more clearly reflecting the relationships between variables [27]. Figure 6 shows the results of PCA analysis for all samples. In this case, the different colors represent different samples of *Rhizoma gastrodiae*, the distance between individual points represents the level of similarity, and the dispersion of the same points represents the homogeneity of the same sample. The results in Figure 6 show that the total contribution of the two principal components, Dim1 and Dim2, was 64.3%. Moreover, the volatile components of the different varieties of *Rhizoma gastrodiae* were significantly different, with the Yunnan Wu Tian Ma and Yunnan Hong Tian Ma samples clustered into one group and the Yunnan Lv Tian Ma showing significant differences from the other samples. The two samples of Yunnan Huang Tian Ma were clustered at a certain distance from each other at the time of harvesting. This also suggests that there were some previous differences between the two.

#### 2.4.2. Fingerprint Similarity Analysis Based on Euclidean Distance

**Figure 7:**
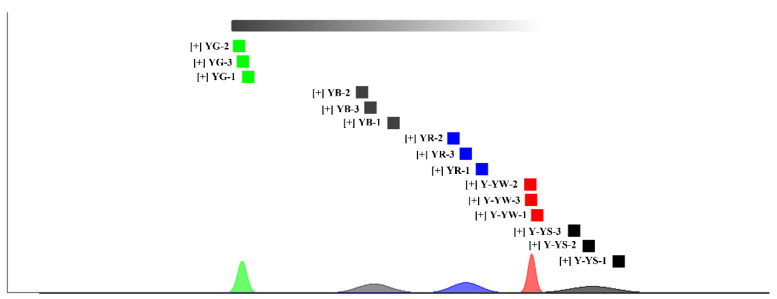
Fingerprint similarity based on the Euclidean distance of different samples.

Euclidean distance is like PCA analysis in that the principle used to determine similarity is the distance coefficient; if the coefficient is large, the difference between the two is also large and shows a positive correlation. Conversely, the smaller the coefficient, the smaller and more similar the difference between the two [28]. The quality of the two samples was evaluated by the Euclidean distance similarity algorithm, and the algorithm was found to be accurate and reliable in evaluating the samples [28]. Figure 7 shows the fingerprint similarity based on Euclidean distance, and Table S1 represents the Euclidean distance values between the five *Rhizoma gastrodiae* samples. From the results of the Euclidean distance analysis, it can be found that the distances between the different species of *Rhizoma gastrodiae* samples can be clearly distinguished. The average Euclidean distance between YG and YB was 14,900,000; the average Euclidean distance between YB and YR was 7,417,990.556; the average Euclidean distance between YR and Y-YW was 5,776,189.222; the average Euclidean distance between Y-YW and Y-YS was 5,332,821.778; and the average Euclidean distance was 28,244,444.444, so the distance between YG and Y-YS was the furthest, i.e., the difference between Yunnan Lv Tian Ma and Yunnan Huang Tian Ma (harvested in spring) was considered to be the most significant. It was also shown that the use of the HS-GC-IMS method was able to distinguish between samples of different species and different harvesting times of *Rhizoma gastrodiae*.

#### 2.4.3. Hierarchical Cluster Analysis Heatmap

**Figure 8:**
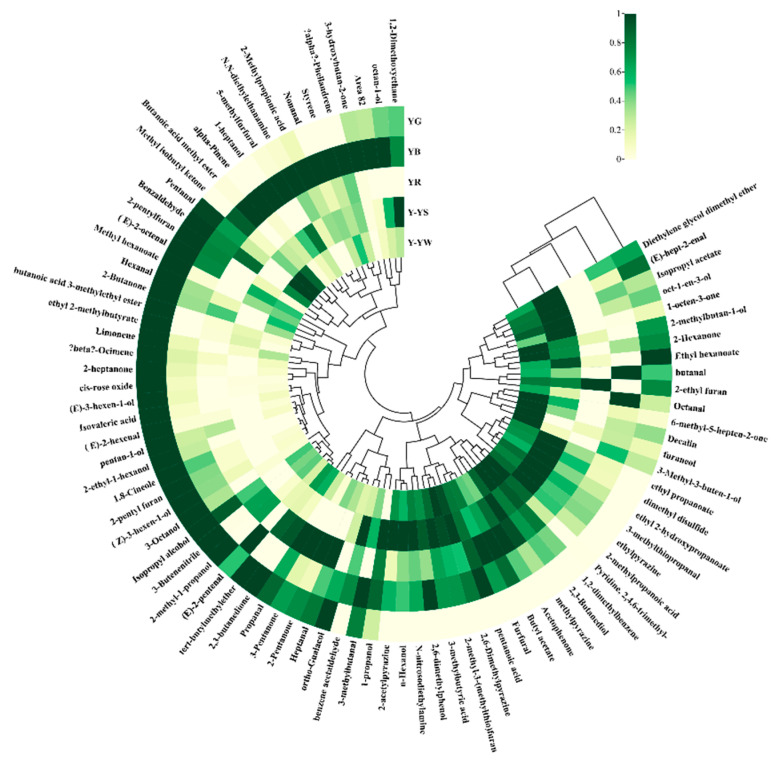
HCA of volatile components in five samples of *Rhizoma gastrodiae*.

To further analyze the differences in VOCs between the different *Rhizoma gastrodiae* samples, a hierarchical cluster analysis (HCA) heatmap was generated. HCA can be used to distinguish between two sample clusters [29] and to analyze the degree of difference in the composition of the samples tested [30,31]. Figure 8 shows the HCA of volatiles in different *Rhizoma gastrodiae* samples. The outer circle represents the volatiles detected, and each column at the opening of the circle indicates the name of each sample. The color indicates a low relative strength, with darker green indicating a stronger strength and a higher content and a lighter color indicating a weaker strength and a lower content. It is clear from Figure 8 that the relative content of volatile substances varied between samples. YG (Yunnan Lv Tian Ma) and YB (Yunnan Wu Tian Ma) samples contained higher and more diverse levels of volatile substances, which were significantly different from the other *Rhizoma gastrodiae* samples. For Huang Tian Ma, the volatile substances were closer in the two harvest times, winter and spring, but winter-harvested *Rhizoma gastrodiae* possessed a higher abundance of volatile substances and species than spring-harvested *Rhizoma gastrodiae*. This study used HS-GC-IMS to analyze VOCs in different *Rhizoma gastrodiae* samples, which has a more convenient operation, a fast response, a high sensitivity, and a low cost.

## 3. Materials and Methods

### 3.1. Sample Preparation

**Figure 9:**
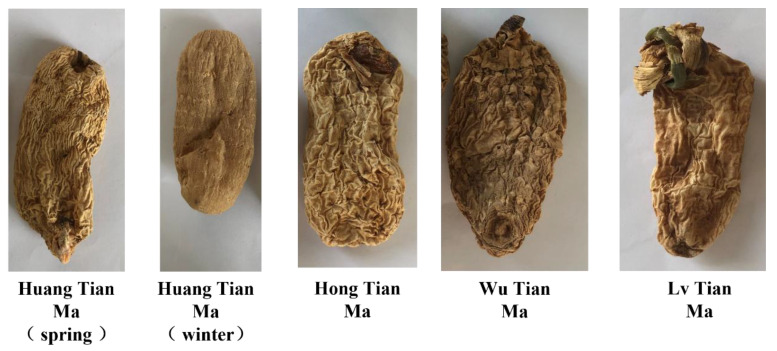
Five varieties of *Rhizoma gastrodiae*.

All Tian Ma samples were produced by Yunnan Zhaotong Xiaocaoba, were provided by Yiliang County Xiaocaoba Wild Tian Ma Co, Ltd., and were dried products. The appearance of the five species of Tian Ma is shown in Figure 9. Yunnan Huang Tian Ma (spring Harvest), Yunnan Huang Tian Ma (winter harvest), Yunnan Huang Tian Ma, Yunnan Wu Tian Ma, and Yunnan Lv Tian Ma were crushed in a pulverizer and filtered through a 24-mesh sieve to obtain the test samples Y-YS, Y-YW, YR, YB, and YG in turn.

### 3.2. HS-GC-IMS System

We used the Gas-phase Ion Mobility Spectrum Flavor Spec^®^ (G.A.S. department of Shandong Province, China; Shandong Hai Neng Science Instrument Co., Ltd.) to analyze six different regions of *Rhizoma gastrodiae* powder prepared previously. We placed 2 g of *Rhizoma gastrodiae* powder in a 20 mL top-empty bottle and incubator for 20 min at 70 °C and 500 rpm under a gas phase temperature. Next, we set the temperature of the injection needle to 85 °C and injected 300 microliters of the sample.

Then, we performed gas chromatography separation using an MXT-5 chromatography column (15 m × 0.53 mm × 1 μm) at a column temperature of 60 °C. We set the IMS temperature to 45 °C and used N_2_ (purity ≥ 99.999%) as the carrier/drift gas, with a flow rate of 2 mL/min (0–2 min), 10 mL/min (2–10 min), 100 mL/min (10–20 min), and 150 mL/min (20–30 min), stopping the analysis after 30 min. The drift tube is maintained at 45 °C under the N_2_ drift gas with a flow rate of 150 mL/min. Three samples are measured for each sample.

### 3.3. Data Analysis

All samples were analyzed in triplicate. GC-IMS library Search software (Version number: 1.0.3) and the Laboratory Analytical Viewer (LAV) are data analysis software (Version number: 2.2.1) that allow different perspectives to be examined. The LAV includes VOCal and three plug-ins, and VOCal is used to view analytical spectra and qualitative and quantitative analysis data. Volatile organic compounds are represented by each point on the graph. With the software’s built-in database, qualitative analyses of substances can be performed. A Reporter plug-in can be used to compare the spectral differences between different products, such as 2D top views and sample difference spectra. Using the library plot plug-in, differences in VOCs between the samples were visually compared using inter-sample fingerprinting.

To facilitate the rapid identification of unknown sample types, the peak volume data of the samples were normalized, the dynamic PCA plug-in was used for the cluster analysis of the samples. A principal component analysis was used to investigate the relationship between different samples and VOCs. Using the clustering heat map tool, heat maps were created.

## 4. Conclusions

In this study, HS-GC-IMS and PCA analyses were used to compare the differences in volatile organic compounds of Yunnan Huang Tian Ma (containing both winter and spring harvesting times), Yunnan Hong Tian Ma, Yunnan Wu Tian Ma, and Yunnan Lv Tian Ma, four species, and two harvesting seasons of *Rhizoma gastrodiae*. A total of 160 signal peaks were identified and 95 compounds were characterized, including 24 aldehydes, 14 ketones, 15 alcohols, 13 heterocyclic compounds, 9 esters, 5 acids, 4 alkenes, 3 hydrocarbons, 3 amines, 2 phenols, 2 ethers, and 1 nitrile. At present, there are still 65 signal peaks that have not been identified, among which 7 components are unique to Wu Tian Ma, 18 components are unique to Lv Tian Ma, and the remaining signal peaks can be detected in all varieties of *Rhizoma gastrodiae*, which once again indicates that there is a richer variety of flavor compounds in Lv Tian Ma. However, from the results of the PCA, Euclidean distance, and hierarchical cluster analysis heatmap, the use of HS-GS-IMS can completely and effectively distinguish the two, and these unknown components can be further characterized and determined with the help of other analytical techniques in the future.

The results of the PCA analysis, cluster analysis based on Euclidean distance, and fingerprint similarity analysis showed that different species of *Rhizoma gastrodiae* contained mainly acetophenone, (E)-2-octenal, 1,2-dimethoxyethane, butanal, 3-methylbutanal, 2-hexanone hexanal, benzaldehyde, 2-methylpropionic acid, alpha-pinene, pyridine,2,4,6-trimethyl-, octanal, benzene acetaldehyde, (E)-hept-2-enal, (E)-hept-2-enal pentanal, nonanal, 2-ethylfuran, heptanal, tert-butylmethylether, isopropyl acetate, isopropyl alcohol, and 2-methylbutan-1-ol. There was some variation between varieties and harvesting seasons, with the YG (Yunnan Lv Tian Ma) and YB (Yunnan Wu Tian Ma) samples containing higher levels of volatile substances, more variety, and better quality. The samples harvested in winter had a higher content and variety of volatile substances compared to those harvested in spring, reflecting the superior quality of the winter-harvested *Rhizoma gastrodiae*. This suggests that differences in the species and harvesting time affect the results of volatile substances in *Rhizoma gastrodiae*. At the same time, we presume that the special smell of *Rhizoma gastrodiae* should be related to its richness in aldehydes, especially benzene acetaldehyde, Benzaldehyde, Heptanal, Hexanal, Pentanal, and butanal, which contain a strong combination of the special smell of aldehydes and the formation of these substances.

In conclusion, HS-GC-IMS was used in this study, which can detect the differences in volatile compounds in different varieties of Yunnan *Rhizoma gastrodiae* samples with different harvesting seasons for rapid analysis in a simple, rapid, and accurate way. The results of this study can help to provide certain references for the screening of raw materials and the improvement of the nutritional value of functional foods of Yunnan Tian Ma.

## Figures and Tables

**Table 1:** Results of the qualitative analysis of five samples of *Rhizoma gastrodiae* (odor description queried at: https://www.femaflavor.org/ (accessed on 15 July 2023).

No.	Compound	CAS#	Formula	MW	RI	Rt [sec]	Dt [a.u.]	Category	Odor	Identification Approach
1	*cis*-rose oxide	C3033236	C_10_H_18_O	154.3	1116.1	862.35	1.36395	Ether	sweet, rose	RI, DT
2	Nonanal ^a^	C124196	C_9_H_18_O	142.2	1085.5	790.725	1.47499	Aldehydes	fat, citrus, green	RI, DT
3	Nonanal ^b^	C124196	C_9_H_18_O	142.2	1085.3	790.299	1.95001	Aldehydes		RI, DT
4	ortho-Guaiacol ^b^	C90051	C_7_H_8_O_2_	124.1	1087	794.136	1.22412	Phenols	burnt, phenol, wood	RI, DT
5	ortho-Guaiacol ^a^	C90051	C_7_H_8_O_2_	124.1	1088.8	798.400	1.12336	Phenols		RI, DT
6	furaneol	C3658773	C_6_H_8_O_3_	128.1	1070.8	756.192	1.20972	Heterocyclic	caramel	RI, DT
7	Acetophenone ^a^	C98862	C_8_H_8_O	120.2	1061.5	734.449	1.18711	Ketones	must, flower, almond	RI, DT
8	Acetophenone ^b^	C98862	C_8_H_8_O	120.2	1064.7	742.123	1.58809	Ketones		RI, DT
9	Decalin	C91178	C_10_H_18_	138.3	1065.8	744.681	1.32899	Hydrocarbons	green, nut, fat	RI, DT
10	(E)-2-octenal ^a^	C2548870	C_8_H_14_O	126.2	1053.1	714.838	1.33105	Aldehydes	/	RI, DT
11	3-sec-Butyl-2- methoxypyrazine	C24168705	C_9_H_14_N_2_O	166.2	1091.5	804.689	1.27823	Heterocyclic	/	RI, DT
12	N-Nitrosodi- N-propylamine	C621647	C_6_H_14_N_2_O	130.2	1076.2	768.858	1.29053	Amines	/	RI, DT
13	(E)-2-octenal ^b^	C2548870	C_8_H_14_O	126.2	1051.5	711.172	1.82123	Aldehydes	green, nut, fat	RI, DT
14	2-ethyl-1-hexanol	C104767	C_8_H_18_O	130.2	1037.3	677.882	1.7773	Alcohols	green, rose	RI, DT
15	beta-Ocimene	C13877913	C_10_H_16_	136.2	1039.7	683.473	1.66659	Alkenes	floral	RI, DT
16	Limonene ^b^	C138863	C_10_H_16_	136.2	1030.1	660.856	1.69119	Alkenes	lemon, orange	RI, DT
17	benzene acetaldehyde	C122781	C_8_H_8_O	120.2	1046.1	698.445	1.25225	Aldehydes	berry, geranium, honey, nut, pungent	RI, DT
18	1.8-Cineole	C470826	C_10_H_18_O	154.3	1039.4	682.639	1.29027	Alcohols	mint, sweet	RI, DT
19	Limonene ^a^	C138863	C_10_H_16_	136.2	1029.8	660.325	1.26249	Alkenes	lemon, orange	RI, DT
20	2-acetylpyrazine	C22047252	C_6_H_6_N_2_O	122.1	1024.3	647.309	1.15281	Heterocyclic	roast	RI, DT
21	Octanal ^a^	C124130	C_8_H_16_O	128.2	1006.8	606.4	1.40141	Aldehydes	citrus, fat, green, oil, pungent	RI, DT
22	Octanal ^b^	C124130	C_8_H_16_O	128.2	1007.5	607.95	1.81965	Aldehydes		RI, DT
23	2,4,5-Trimethylthiazole	C13623115	C_6_H_9_NS	127.2	999.5	589.355	1.14912	Heterocyclic	cocoa, earthy, nut	RI, DT
24	Pyridine, 2,4,6- trimethyl-	C108758	C_8_H_11_N	121.2	988.9	567.97	1.16408	Heterocyclic	/	RI, DT
25	2-pentyl furan	C3777693	C_9_H_14_O	138.2	994.2	577.962	1.25391	Heterocyclic	butter, floral, fruit, green bean	RI, DT
26	3-Octanol^b^	C589980	C_8_H_18_O	130.2	998.1	586.088	1.76519	Alcohols	moss, nut, mushroom	RI, DT
27	1-octen-3-one ^a^	C4312996	C_8_H_14_O	126.2	980.3	551.638	1.27132	Ketones	earth, mushroom	RI, DT
28	Ethyl hexanoate	C123660	C_8_H_16_O_2_	144.2	1000.2	590.895	1.3385	Esters	apple peel, fruit	RI, DT
29	1-octen-3-one ^b^	C4312996	C_8_H_14_O	126.2	980.2	551.409	1.69428	Ketones	earth, mushroom	RI, DT
30	oct-1-en-3-ol	C3391864	C_8_H_16_O	128.2	982.5	555.758	1.59352	Alcohols	cucumber, earth, fat, floral, mushroom	RI, DT
31	(E)-hept-2-enal ^b^	C18829555	C_7_H_12_O	112.2	956.9	507.516	1.67599	Aldehydes	almond, fat, fruit	RI, DT
32	(E)-hept-2-enal ^a^	C18829555	C_7_H_12_O	112.2	956.2	506.066	1.26737	Aldehydes		RI, DT
33	Benzaldehyde ^a^	C100527	C_7_H_6_O	106.1	969.3	530.943	1.14967	Aldehydes	almond, burnt sugar	RI, DT
34	Benzaldehyde ^b^	C100527	C_7_H_6_O	106.1	968.6	529.525	1.47168	Aldehydes		RI, DT
35	5-Methylfurfural	C620020	C_6_H_6_O_2_	110.1	954.9	503.755	1.47723	Heterocyclic	almond, caramel, burnt sugar	RI, DT
36	1-heptanol	C111706	C_7_H_16_O	116.2	961.9	516.995	1.38507	Alcohols	chemical, green	RI, DT
37	Ethylpyrazine ^b^	C13925003	C_6_H_8_N_2_	108.1	930.5	457.601	1.15078	Heterocyclic	burnt, green, iron scorch, must, peanut butter, roasted, rum, wood	RI, DT
38	Ethylpyrazine ^a^	C13925003	C_6_H_8_N_2_	108.1	929.5	455.774	1.11192	Heterocyclic		RI, DT
39	3-methylthiopropanal	C3268493	C_4_H_8_O_S_	104.2	912.8	424.181	1.0886	Aldehydes	cooked potato, soy	RI, DT
40	2,6-Dimethylpyrazine	C108509	C_6_H_8_N_2_	108.1	913.9	426.27	1.14967	Heterocyclic	cocoa, coffee, green, roast beef, roasted nut	RI, DT
41	Methyl hexanoate	C106707	C_7_H_14_O_2_	130.2	921.2	440.108	1.28846	Esters	fruit, fresh, sweet	RI, DT
42	Heptanal ^b^	C111717	C_7_H_14_O	114.2	900.3	400.683	1.69819	Aldehydes	fat, citrus, rancid	RI, DT
43	3-Octanol ^a^	C589980	C_8_H_18_O	130.2	988.3	566.739	1.39173	Alcohols	moss, nut, mushroom	RI, DT
44	2-heptanone ^b^	C110430	C_7_H_14_O	114.2	890.5	383.379	1.63571	Ketones	soap	RI, DT
45	Heptanal ^a^	C111717	C_7_H_14_O	114.2	901.7	403.294	1.32286	Aldehydes	fat, citrus, rancid	RI, DT
46	n-Hexanol	C111273	C_6_H_14_O	102.2	867.5	357.353	1.32385	Alcohols	banana, flower, grass, herb	RI, DT
47	pentanoic acid	C109524	C_5_H_10_O_2_	102.1	896.5	393.484	1.22546	Acids	sweat	RI, DT
48	2-heptanone ^a^	C110430	C_7_H_14_O	114.2	894	388.698	1.26285	Ketones	soap	RI, DT
49	N-nitrosodiethylamine	C55185	C_4_H_10_N_2_O	102.1	896.2	392.766	1.1497	Amines	/	RI, DT
50	1,2-dimethylbenzene	C95476	C_8_H_10_	106.2	863.3	352.567	1.0828	Hydrocarbons	/	RI, DT
51	Furfural	C98011	C_5_H_4_O_2_	96.1	846.8	333.904	1.08378	Aldehydes	bread, almond, sweet	RI, DT
52	methylpyrazine	C109080	C_5_H_6_N_2_	94.1	827.3	311.89	1.09461	Heterocyclic	cocoa, green, hazelnut, popcorn, roasted	RI, DT
53	(E)-2-hexenal	C6728263	C_6_H_10_O	98.1	848.3	335.578	1.18414	Aldehydes	apple, green	RI, DT
54	3-methylbutyric acid	C503742	C_5_H_10_O_2_	102.1	829	313.804	1.21956	Acids	cheese, pungent	RI, DT
55	butanoic acid 3- methylethyl ester	C108645	C_7_H_14_O_2_	130.2	857.6	346.107	1.26186	Esters	apple, fruit, pineapple, sour	RI, DT
56	ethyl 2-methylbutanoate	C7452791	C_7_H_14_O_2_	130.2	858.2	346.824	1.63966	Esters	apple, ester, green apple, kiwi, strawberry	RI, DT
57	(E)-3-hexen-1-ol	C928972	C_6_H_12_O	100.2	847	334.143	1.5216	Alcohols	green	RI, DT
58	Isovaleric acid	C503742	C_5_H_10_O_2_	102.1	834.3	319.786	1.48913	Acids	sweat, acid, rancid	RI, DT
59	ethyl 2-hydroxypropanoate	C97643	C_5_H_10_O_3_	118.1	819.3	302.785	1.15121	Esters	cheese, floral, fruit, pungent, rubber	RI, DT
60	Hexanal ^b^	C66251	C_6_H_12_O	100.2	802.2	283.496	1.57059	Aldehydes	grass, tallow, fat	RI, DT
61	2-Hexanone	C591786	C_6_H_12_O	100.2	792.1	272.015	1.50704	Ketones	/	RI, DT
62	2,3-Butanediol	C513859	C4H_10_O_2_	90.1	809.1	291.304	1.35878	Alcohols	fruit, onion	RI, DT
63	2-Methylpropionic acid	C79312	C_4_H_8_O_2_	88.1	789.4	268.916	1.35454	Acids	burnt, butter, cheese, sweat	RI, DT
64	Hexanal ^a^	C66251	C_6_H_12_O	100.2	803.5	284.874	1.2557	Aldehydes	grass, tallow, fat	RI, DT
65	Ethyl butyrate	C105544	C_6_H_12_O_2_	116.2	786.6	265.76	1.21954	Esters	apple	RI, DT
66	pentan-1-ol ^a^	C71410	C_5_H_12_O	88.1	775	255.54	1.25267	Alcohols	balsamic, fruit, green, pungent, yeast	RI, DT
67	pentan-1-ol ^b^	C71410	C_5_H_12_O	88.1	772.9	253.856	1.52068	Alcohols		RI, DT
68	2-methylbutan-1-ol	C137326	C_5_H_12_O	88.1	748.8	234.202	1.24213	Alcohols	fish oil, green, malt, onion, wine	RI, DT
69	N,N-diethylethanamine	C121448	C_6_H_15_N	101.2	709.1	201.858	1.21653	Amines	/	RI, DT
70	Hydroxyacetone	C116096	C_3_H_6_O_2_	74.1	721.8	212.19	1.21352	Ketones	butter, herb, malt, pungent	RI, DT
71	2-methylpropanoic acid	C79312	C_4_H_8_O_2_	88.1	782.8	261.942	1.14576	Acids	burnt, butter, cheese, sweat	RI, DT
72	(E)-2-pentenal	C1576870	C_5_H_8_O	84.1	748.8	234.202	1.35656	Aldehydes	/	RI, DT
73	Butanoic acid methyl ester	C623427	C_5_H_10_O_2_	102.1	730.3	219.153	1.44389	Esters	apple, banana, cheese, ester, floral	RI, DT
74	Acetoin	C513860	C_4_H_8_O_2_	88.1	735	222.971	1.34451	Ketones	butter, cream	RI, DT
75	3-Methyl-3-buten-1-ol	C763326	C_5_H_10_O	86.1	721.5	211.965	1.28579	Alcohols	/	RI, DT
76	Pentanal	C110623	C_5_H_10_O	86.1	695	190.403	1.43034	Aldehydes	almond, malt, pungent	RI, DT
77	ethyl propanoate	C105373	C_5_H_10_O_2_	102.1	712.7	204.778	1.14576	Esters	apple, pineapple, rum, strawberry	RI, DT
78	Methyl isobutyl ketone	C108101	C_6_H_12_O	100.2	729.8	218.704	1.48454	Ketones	/	RI, DT
79	2,5-Dimethylfuran	C625865	C_6_H_8_O	96.1	705.4	198.826	1.33548	Heterocyclic	savory	RI, DT
80	2-Ethylfuran	C3208160	C_6_H_8_O	96.1	697.5	192.409	1.28502	Heterocyclic	butter, caramel	RI, DT
81	3-methylbutanal ^b^	C590863	C_5_H_10_O	86.1	650.1	168.205	1.40884	Aldehydes	malt	RI, DT
82	1,2-Dimethoxyethane	C110714	C_4_H_10_O_2_	90.1	646.3	166.547	1.31833	Hydrocarbons	/	RI, DT
83	pentan-2-one	C107879	C_5_H_10_O	86.1	675	179.146	1.11851	Ketones	fruit, pungent	RI, DT
84	Isopropyl acetate	C108214	C_5_H_10_O_2_	102.1	667	175.665	1.1612	Esters	banana	RI, DT
85	3-Butenenitrile	C109751	C_4_H_5_N	67.1	633.5	160.911	1.11851	Nitrile	/	RI, DT
86	3-methylbutanal ^a^	C590863	C_5_H_10_O	86.1	643.3	165.221	1.15181	Aldehydes	malt	RI, DT
87	3-Pentanone	C96220	C_5_H_10_O	86.1	688.8	185.28	1.33114	Ketones	/	RI, DT
88	2-methyl-1- propanol	C78831	C_4_H_10_O	74.1	622.9	156.228	1.17831	Alcohols	apple, bitter, cocoa, wine	RI, DT
89	Butanal ^a^	C123728	C_4_H_8_O	72.1	608.2	149.753	1.12118	Aldehydes	pungent, green	RI, DT
90	Butanal ^b^	C123728	C_4_H_8_O	72.1	598.2	145.366	1.29119	Aldehydes		RI, DT
91	2-Butanone	C78933	C_4_H_8_O	72.1	590.9	142.129	1.24938	Ketones	fragrant, fruit, pleasant	RI, DT
92	2,3-butanedione	C431038	C_4_H_6_O_2_	86.1	589.7	141.606	1.1818	Ketones	butter, pastry, yeast	RI, DT
93	tert-butyl methyl ether	C1634044	C_5_H_12_O	88.1	548.4	123.434	1.11769	Ether	/	RI, DT
94	Isopropyl alcohol	C67630	C_3_H_8_O	60.1	507.1	105.262	1.08754	Alcohols	floral	RI, DT
95	alpha-Pinene	C80568	C_10_H_16_	136.2	937.2	470.198	1.21549	Alkenes	cedarwood, pine, sharp	RI, DT

Note: MW: molecular mass; RI: retention index; Rt: retention time; Dt: drift time; ^a^: monomer; ^b^: dimer.

## Data Availability

The data are contained within the article and Supplementary Materials.

## Supplementary Materials

The following supporting information can be downloaded at: https://www.mdpi.com/article/10.3390/molecules28186705/s1, Table S1. Euclidean distances between different *Rhizoma gastrodiae* species.

## Sample Availability

Samples of the compounds are not available from the authors.

## References

[1] Zhou X., Chen X.Q. (1983). The collation of the genus Amanita in China. Yunnan Plant Res..

[2] Duan H., Zhou Y., Zhou S., Yan W. (2023). Application of asparagus in health food in China. Food Ind. Sci. Technol..

[3] Fan Q., Chen C., Huang Z., Zhang C., Liang P., Zhao S. (2015). Discrimination of Rhizoma gastrodiae (Tianma) using 3D synchronous fluorescence spectroscopy coupled with principal component analysis. Spectrochim. Acta Part A Mol. Bio-Mol. Spectrosc..

[4] Zhan H.D., Zhou H.Y., Sui Y.P., Du X.L., Wang W.H., Dai L., Sui F., Huo H.R., Jiang T.L. (2016). The rhizome of Gastrodia elata Blume—An ethnopharma-cological review. J. Ethnopharmacol..

[5] Liu Y., Gao J., Peng M., Meng H., Ma H., Cai P., Xu Y., Zhao Q., Si G. (2018). A Review on Central Nervous System Effects of Gastrodin. Front. Pharmacol..

[6] Zhang J., Li L., Liu Q., Zhao Z., Su D., Xiao C., Jin T., Chen L., Xu C., You Z. (2023). Gastrodin programs an Arg-1^+^ microglial phenotype in hippocampus to ameliorate depression- and anxiety-like behaviors via the Nrf2 pathway in mice. Phytomedicine.

[7] Cheng L., Wang H., Ma K., Deng Y., Li M., Ma J. (2023). A novel alcohol steamed preparation from Gastrodia elata Blume: Pharmaco-logical assessment of a functional food. Front. Pharmacol..

[8] Sun H., Hao D., Li X., Jin W. (2022). Analysis of the variability of volatile substances in fresh asparagus of different varieties and origins. Food Mach..

[9] Gonçalves W.B., Teixeira W.S., Cervantes E.P., Mioni M.D., Sampaio A.N., Martins O.A., Gruber J., Pereira J.G. (2023). Application of an Electronic Nose as a New Technology for Rapid Detection of Adulteration in Honey. Appl. Sci..

[10] Capitain C., Weller P. (2021). Non-Targeted Screening Approaches for Profiling of Volatile Organic Compounds Based on Gas Chromatography-Ion Mobility Spectroscopy (GC-IMS) and Machine Learning. Molecules.

[11] Kiani S., Minaei S., Ghasemi-Varnamkhasti M. (2017). Integration of computer vision and electronic nose as non-destructive systems for saffron adulteration detection. Comput. Electron. Agric..

[12] Wang S., Chen H., Sun B. (2020). Recent progress in food flavor analysis using gas chromatography–ion mobility spectrometry (GC–IMS). Food Chem..

[13] Slimani S., Bultel E., Cubizolle T., Herrier C., Rousselle T., Livache T. (2020). Opto-Electronic Nose Coupled to a Silicon Micro Pre-Concentrator Device for Selective Sensing of Flavored Waters. Chemosensors.

[14] Rasekh M., Karami H., Wilson A.D., Gancarz M. (2021). Performance Analysis of MAU-9 Electronic-Nose MOS Sensor Array Com-ponents and ANN Classification Methods for Discrimination of Herb and Fruit Essential Oils. Chemosensors.

[15] Yin J., Wu M., Lin R., Li X., Ding H., Han L., Yang W., Song X., Li W., Qu H. (2021). Application and development trends of gas chromatography–ion mobility spectrometry for traditional Chinese medicine, clinical, food and environmental analysis. Microchem. J..

[16] Chen D., Qin L., Geng Y., Kong Q., Wang S., Lin S. (2021). The Aroma Fingerprints and Discrimination Analysis of Shiitake Mushrooms from Three Different Drying Conditions by GC-IMS, GC-MS and DSA. Foods.

[17] Wang J., Li X., Wu Y., Qu F., Liu L., Wang B., Wang P., Zhang X. (2022). HS−SPME/GC−MS Reveals the Season Effects on Volatile Compounds of Green Tea in High−Latitude Region. Foods.

[18] Ning M., Rui G., Rentang Z., Yue G. (2022). GC-IMS-Based Preliminary Analysis of Volatile Flavor Compounds in Ejiao at Different Processing Stages. J. Food Qual..

[19] Di W., Jian Z., Zongshuai Z., Yang L., Suhong H., Ming H. (2021). Effect of ageing time on the flavour compounds in Nanjing wa-ter-boiled salted duck detected by HS-GC-IMS. LWT—Food Sci. Technol..

[20] Zhang J., Zhang W., Zhou L., Zhang R. (2021). Study on the influences of ultrasound on the flavor profile of unsmoked bacon and its underlying metabolic mechanism by using HS-GC-IMS. Ultrason. Sonochem..

[21] Qiu H., Zhou X., Wu L., Yao M., Shen X., Xiao T., Xu Q., Tao L. (2019). Headspace gas chromatography analysis of volatile components of asparagus. Zizhen Guoji Guomao.

[22] Cao S., Zhao C.F., Ma F.W., Ma C., Li Y., Wang R. (2019). Evaluation of aromatic quality of asparagus at different harvesting stages based on e-nose and GC-MS. North. Hort..

[23] Lu Y. (2016). Analysis of Volatile Components of Asparagus and Walnut and Development of Their Products. Master’s Thesis.

[24] Huang M.Z., Li X. (2018). Analysis of the species and content of volatile components of asparagus by SDE-GC-MS. J. Guizhou Agric. Sci..

[25] Chen L., Li X., Li Z., Deng L. (2020). Analysis of 17 elements in cow, goat, buffalo, yak, and camel milk by inductively coupled plasma mass spectrometry (ICP-MS). RSC Adv..

[26] Jia W., Zhang R., Shi L., Zhang F., Chang J., Chu X. (2019). Accurate determination of volatile-flavor components in bos grunniens milk by high-throughput dynamic headspace gas chromatographic-mass spectrometry. J. Chromatogr. A.

[27] Christmann J., Rohn S., Weller P. (2022). gc-ims-tools—A new Python package for chemometric analysis of GC–IMS data. Food Chem..

[28] Zhou S.Q., Feng D., Zhou Y.X., Zhao J., Zhao J.Y., Guo Y., Yan W.J. (2022). HS-GC-IMS detection of volatile organic compounds in cistanche powders under different treatment methods. LWT—Food Sci. Technol..

[29] Duan Z., Dong S., Dong Y., Gao Q. (2021). Geographical origin identification of two salmonid species via flavor compound analysis using headspace-gas chromatography-ion mobility spectrometry combined with electronic nose and tongue. Food Res. Int..

[30] Lu W., Chen J., Li X., Qi Y., Jiang R. (2023). Flavor components detection and discrimination of isomers in Huaguo tea using head-space-gas chromatography-ion mobility spectrometry and multivariate statistical analysis. Anal. Chim. Acta.

[31] Liu A., Zhang H., Liu T., Gong P., Wang Y., Wang H., Tian X., Liu Q., Cui Q., Xie X. (2022). Aroma classification and flavor characterization of Streptococcus thermophilus fermented milk by HS-GC-IMS and HS-SPME-GC-TOF/MS. Food Biosci..

