# Supplementary material for: HS-GC-IMS Analysis of Volatile Organic Compounds in Different Varieties and Harvesting Times of Rhizoma gastrodiae (Tian Ma) in Yunnan Province
Source: Molecules. 2023 Sep 20;28(18):6705. doi: 10.3390/molecules28186705 (PMC10536806; doi:10.3390/molecules28186705)
Supplement: Supplementary file 1 [file molecules-28-06705-s001.zip › molecules-2535023-supplementary.pdf]

Table **S1.** Euclidean distances between different *Rhizoma gastrodiae* species.

**Full distance**	**[+] Y-YS-1**	**[+] Y-YS-2**	**[+] Y-YS-3**	**[+] Y-YW-1**	**[+] Y-YW-2**	**[+] Y-YW-3**
[+] Y-YS-1	0	3457994	5329298	7094685	7055707	7058979
[+] Y-YS-2	3457994	0	1663898	5521527	4670980	5117810
[+] Y-YS-3	5329298	1663898	0	4224954	3576892	3673862
[+] Y-YW-1	7094685	5521527	4224954	0	731500.9	720989.3
[+] Y-YW-2	7055707	4670980	3576892	731500.9	0	131816.1
[+] Y-YW-3	7058979	5117810	3673862	720989.3	131816.1	0
[+] YR-1	8318072	8647970	8064405	6822527	5570357	5747812
[+] YR-2	8145442	7046840	7108445	5390898	5143419	5662572
[+] YR-3	8922799	7280242	7541566	6923450	5106019	5618649
[+] YB-1	1.02E+07	1.04E+07	9497628	6543579	6654086	6428196
[+] YB-2	1.08E+07	1.04E+07	1.02E+07	7128638	7068813	7154383
[+] YB-3	1.28E+07	1.20E+07	1.16E+07	7306260	8775925	8841816
[+] YG-1	2.65E+07	2.71E+07	2.68E+07	2.21E+07	2.16E+07	2.15E+07
[+] YG-2	2.95E+07	3.03E+07	3.01E+07	2.47E+07	2.49E+07	2.49E+07
[+] YG-3	2.74E+07	2.83E+07	2.82E+07	2.32E+07	2.30E+07	2.29E+07

(continued)

**[+] YR-1**	**[+] YR-2**	**[+] YR-3**	**[+] YB-1**	**[+] YB-2**	**[+] YB-3**	**[+] YG-1**	**[+] YG-2**	**[+] YG-3**
8318072	8145442	8922799	1.02E+07	1.08E+07	1.28E+07	2.65E+07	2.95E+07	2.74E+07
8647970	7046840	7280242	1.04E+07	1.04E+07	1.20E+07	2.71E+07	3.03E+07	2.83E+07
8064405	7108445	7541566	9497628	1.02E+07	1.16E+07	2.68E+07	3.01E+07	2.82E+07

6822527	5390898	6923450	6543579	7128638	7306260	2.21E+07	2.47E+07	2.32E+07
5570357	5143419	5106019	6654086	7068813	8775925	2.16E+07	2.49E+07	2.30E+07
5747812	5662572	5618649	6428196	7154383	8841816	2.15E+07	2.49E+07	2.29E+07
0	2052835	1841558	6397785	6845389	9718460	1.84E+07	2.18E+07	1.93E+07
2052835	0	1441542	6650700	6414245	6880967	1.82E+07	2.05E+07	1.88E+07
1841558	1441542	0	7208268	6889797	9756304	1.84E+07	2.18E+07	1.94E+07
6397785	6650700	7208268	0	978076.5	2703248	1.33E+07	1.58E+07	1.42E+07
6845389	6414245	6889797	978076.5	0	1738210	1.30E+07	1.60E+07	1.45E+07
9718460	6880967	9756304	2703248	1738210	0	1.49E+07	1.65E+07	1.59E+07
1.84E+07	1.82E+07	1.84E+07	1.33E+07	1.30E+07	1.49E+07	0	1164802	622257.5
2.18E+07	2.05E+07	2.18E+07	1.58E+07	1.60E+07	1.65E+07	1164802	0	478228.5
1.93E+07	1.88E+07	1.94E+07	1.42E+07	1.45E+07	1.59E+07	622257.5	478228.5	0
